# Effects of Zinc (Zn) from Different Sources on Production Performance, Health Status, Antioxidant Properties and Immune Regulation of Dairy Cows in Early Lactation

**DOI:** 10.3390/vetsci12060545

**Published:** 2025-06-03

**Authors:** Xue Li, Jianfei Wang, Maocheng Jiang, Yongjiu Huo, Kang Zhan

**Affiliations:** 1 College of Animal Science and Technology, Yangzhou University, Yangzhou 225009, China; lx929492@163.com; 2 Hunan DeBon Bio-Tech Co., Ltd., Hengyang 421500, China; wangjf_debon_tech@163.com; 3 College of Animal Science and Technology, Anhui Agricultural University, Hefei 230036, China; jmcheng1993@163.com

**Keywords:** Zn, production performance, health status, immune regulation, early lactating cows

## Abstract

This study aimed to investigate the effects of various Zn sources on the performance, health, antioxidant activity, and immune response in early lactation dairy cows. Thirty Holstein cows with similar physiological conditions were randomly assigned to one of three treatment groups, each receiving a distinct supplement: Zn sulfate (CON), Zn amino acid complex (Zn-AA), or Zn methionine complex (Zn-Met), at a dosage of 60 mg/kg on a dry matter basis. The experiment lasted for 67 days, including a 7-day pre-feeding period. The results indicated that supplementation with Zn-AA and Zn-Met significantly reduced the milk somatic cell count compared to the control group (*p* = 0.001). Interestingly, glutathione peroxidase (GSH-PX) levels were markedly increased in both the Zn-AA and Zn-Met groups relative to the control group (*p* = 0.003), with Zn-AA also demonstrating a significant increase in catalase (CAT) content (*p* = 0.001). Both Zn-AA and Zn-Met led to significant elevations in IgA and IgM content (*p* < 0.05). Furthermore, milk Zn content in the Zn-Met groups was significantly elevated than that compared with the control group (*p* < 0.001). In conclusion, organic Zn supplementation resulted in a reduction in the somatic cell count, alongside notable increases the immunoglobulins, milk Zn content, and GSH-PX levels. This study suggests that organic Zn sources, particularly the Zn-AA, are more effective for enhancing the health of dairy cows.

## Simple Summary

Zn is an essential trace element for dairy cows, playing a crucial role in their growth and reproduction, as well as enhancing antioxidant and immune functions. Currently, the effects of Zn during early lactation in dairy cows warrant further investigation. The results of the study indicated that, compared to the control group, both Zn–amino acid (Zn-AA) and Zn–methionine (Zn-Met) significantly improved blood antioxidant capacity and immune function. Furthermore, research has demonstrated that Zn-AA and Zn-Met are more effective than the inorganic Zn in reducing the somatic cell count in milk. In conclusion, organic Zn, particularly in the form of Zn-AA, offers superior supplementation benefits for dairy cows compared to inorganic Zn.

## 1. Introduction

In the early stages of lactation, the significant increase in milk production and the metabolic rate of the mammary gland lead to enhanced aerobic respiration in mammary gland cells. This heightened metabolic activity can result in the excessive production of free radicals, placing the mammary gland under oxidative stress. Consequently, this situation raises important questions regarding the feeding and management of lactating dairy cows, presenting a substantial challenge [1]. To enhance the health of dairy cows, high-quality forage is typically provided, along with the addition of trace elements. The supplementation of trace elements in dairy cow diets has increasingly garnered the attention of scholars [2,3]. Numerous previous animal studies have demonstrated that Zn supplementation can positively influence animal production performance [4], blood parameters [5], blood antioxidant capacity [6], and immunity [7]. Therefore, it is crucial to incorporate trace element Zn into dairy cow diets, as Zn is a key element in enhancing their health and productivity [8].

Zn plays a crucial regulatory role in the formation, development, and immune function of the animal immune system [9]. It enhances the body’s immune response by influencing cellular immunity, humoral immunity, cytokine secretion, and the regulation of gene expression and lymphocyte apoptosis. As a key trace element in immune regulation, Zn is vital for dairy cows, as it maintains the body’s defense mechanisms by activating antioxidant enzymes and regulating immune cell function [10,11]. Notably, Zn deficiency can result in diminished neutrophil activity, compromised mucosal barriers, and an increased susceptibility to mastitis and hoof diseases [12,13]. Dietary supplementation of Zn in dairy cows can elevate immunoglobulin levels and improve anti-inflammatory capacities. Xuejun Zhao et al. [14] reported that incorporating chelated Zn into dairy cow feed enhances their antioxidant status and immune response. Alhussien et al.’s research indicated that Zn supplementation increases IgG and neutrophil levels, thereby improving the humoral immune response in dairy cows [15]. Additionally, findings from LM Nemec’s study suggest that the addition of chelated Zn can bolster the immune response in dairy cows during early lactation [16]. Recent studies underscore the significance of incorporating Zn into feed to meet the dietary requirements of dairy cows and to effectively manage their immune health [17].

Different sources of Zn supplementation exist, such as inorganic sources like Zn sulfate (ZnSO_4_) and organic sources like amino acid–complexed Zn and methionine–chelated Zn [18,19]. Research suggests that amino acid–chelated Zn and methionine–chelated Zn exhibit higher biological efficacy, utilization, and stability compared to Zn sulfate, leading to improved growth performance and reduced environmental pollution [20,21]. The NRC (2021) recommends a dietary Zn amount of 52.8 ppm for lactating dairy cows [22]. However, it has been noted that for dairy cows with continuously improving genetics and increasing yields, supplementing minerals at the NRC-recommended level may be insufficient [23]. While there is ample experimental data on the Zn requirements of dairy cows, there is a lack of research specifically comparing the effects of inorganic Zn and organic Zn supplementation at the same levels in lactating dairy cows.

Therefore, this study aimed to investigate the effects of various forms of Zn supplementation (ZnSO_4_, Zn-AA, and Zn-Met) on the production performance, health status, antioxidant properties, and immune regulation of lactating dairy cows.

## 2. Materials and Methods

All Holstein cows utilized in this research were meticulously cared for in accordance with the principles established by the Institutional Animal Care and Use Committee (IACUC) of Yangzhou University (SYXK (Su) 2016-0019).

### 2.1. Animal Experimental Design and Diet

The Zn sulfate (with a Zn content of 35%), amino acid–Zn complex (with a Zn content of 15%), and methionine–Zn complex (with a Zn content of 19%) utilized in this experiment were supplied by Hunan DeBon Bio-Tech Co., Ltd. Hengyang, China. The experiment was conducted at the Yangzhou University Experimental Farm. A total of thirty healthy Holstein cows, characterized by similar milk yield (32.11 ± 5.03 kg), lactation days (38.48 ± 14.84 days), and parity (2 ± 0.6), were selected for this experiment. Based on factors including milk yield, parity, and lactation days, the cows were randomly assigned to either the control group or the treatment group, with ten cows in each group. This randomization ensured that there were no significant differences in age, lactation days, and initial body condition scores among the groups. The cows were housed in separate stalls to facilitate the monitoring of their feed intake. A total mixed ration (TMR) was formulated in accordance with NRC (Nutrition 2021) standards to fulfill the daily nutritional requirements of lactating cows. Table 1 presents the feed ingredients along with their chemical composition. With the exception of the zinc source, the composition of the TMR diet was consistent across all treatment groups.

The concentration of Zn in the diets for all three groups was 60 mg/kg on a dry matter basis. The experiment lasted for 67 days, which included a 7-day pre-test period. Animals were provided with total mixed rations (TMRs) and had unrestricted access to water, receiving 105% of ad libitum feed intake three times daily at 07:00, 13:00, and 20:00. Additionally, cows were milked three times a day.

### 2.2. Sample Collection and Analysis

Dry matter intake (DMI) of early lactation cows was assessed throughout the trial by continuously recording feed intake and residuals on the last 3 days of each week [24]. Samples collected were subsequently dried in a 65 °C oven (DHG-9240A, Shanghai Jing Hong Laboratory Instrument Co., Ltd., Shanghai, China) for 48 hours, ground using a Wiley grinder (CM100, Beijing Gladman Instrument Co., Ltd., Beijing, China) with a 2 mm sieve, and stored for further analysis.

The AOAC International 2005 guidelines were adhered to for the analysis of dry matter (method 930.15), ash (method 942.05.15), and ether extract (method 996) of the crushed feed [25]. Neutral detergent fiber (NDF) and acid detergent fiber (ADF) were quantified using a fiber analyzer (ANKOM, 2000I, New York, NY, USA) in accordance with the method outlined by Van Soest [26]. The calcium and phosphorus content were determined using the calcium kit (microplate method) (C004-2-1, Nanjing Jian Cheng Bioengineering Institute, Nanjing, China) and the phosphorus kit (phosphomolybdate method) (Cat. No.: C006-1-1, Nanjing Jian Cheng Bioengineering Institute, Nanjing, China), respectively.

The calculation of the fat-corrected milk (FCM) and feed conversion rate (FCR) was performed using the formulas FCM = 0.4 × milk production + 15 × fat yield and FCR = milk production (kg)/dry matter intake (kg) [27].

Milk production was recorded daily, with two 50 mL samples collected from three consecutive milkings on one day each week at 07:30, 14:30, and 21:00 hours. The first portion of each sample was placed in test tubes containing a preservative (0.05% benzoic acid) and submitted to Dairy One Cooperative Inc. (Shanghai, China) for analysis of the milk protein percentage, butterfat percentage, lactose, total solids (TSs), and somatic cell count (SCC) [28]. The second sample, which contained no added preservatives, was used to measure the Zn content in the milk and was analyzed using an inductively coupled plasma spectrometer (Optima 7300 DV, PerkinElmer Inc., Shelton, CT, USA) [29].

Before the 60th day of the experiment, blood samples were collected from the tail vein using a disposable blood collection needle and a vacuum plasma tube (Vacutainer; Becton Dickinson, Franklin Lakes, Nanjing, China). The plasma tube was inverted to mix the contents, followed by centrifugation at 4 °C and 3000 rpm for 15 min to separate the plasma supernatant. The supernatant was then pipetted and stored at −80 °C for subsequent analysis. Plasma samples were sent to Jiangsu Heng Yi Biotechnology Co., Ltd. (Hangzhou, China), for testing. An automatic biochemical analyzer (Mindray; BS-420) was employed to analyze total protein (TP), albumin (ALB), globulin (GLO), glucose (GLU), triglyceride (TG), urea (UREA), alkaline phosphatase (ALP), alanine aminotransferase (ALT), aspartate aminotransferase (AST), γ-glutamyl transferase (γ-GT), total bilirubin (T-BIL), choline esterase (CHE), creatinine (CREA-S), uric acid (UA), total cholesterol (TC), non-esterified fatty acids (NEFA), lactic acid (LAC), and insulin (INS). Total antioxidant capacity (T-AOC), malondialdehyde (MDA), catalase (CAT), and superoxide dismutase (SOD) were analyzed using ELISA kits from the Beijing Sino-British Institute of Biotechnology (Beijing, China). Additionally, glutathione peroxidase (GSX-PX), immunoglobulin A (IgA), immunoglobulin G (IgG), and immunoglobulin M (IgM) were assessed. Tumor necrosis factor-α (TNF-α), interleukin-1β (IL-1β), and interleukin-6 (IL-6) levels in plasma were measured using enzyme-linked immunosorbent assay kits provided by Jiangsu Mei Mian Industrial Co., Ltd. (Yancheng, China). Furthermore, the Zn ion detection kit from Nanjing Jian Cheng Bioengineering Institute (Jiangsu Province) was employed to quantify Zn content in plasma.

Although the Zn source and funding for this study were provided by Hunan DeBon Bio-Tech Co., the research team maintained complete independence in experimental design, sample processing, data analysis, and conclusion derivation. All laboratory procedures adhered to standardized protocols, and blood and milk samples were tested by a third-party laboratory to mitigate potential bias.

### 2.3. Statistical Analysis

The results of the test data are presented as the mean and standard error of the mean. Statistical analysis was conducted using SPSS version 20.0 (IBM Corp., Armonk, NY, USA; SPSS Inc., Chicago, IL, USA). Significant differences were determined through one-way analysis of variance followed by the Tukey multiple comparison test [30]. A significance level of *p* < 0.05 indicates a statistically significant difference, while *p* < 0.01 denotes a highly significant difference. 

## 3. Results

### 3.1. Production Performance

As shown in Table 2, both the Zn-AA and Zn-Met groups have no significant difference in DMI and milk component compared to the control group (*p* > 0.05). Remarkably, the inclusion of Zn-AA and Zn-Met resulted in a significant reduction in somatic cell counts in milk relatively to the control group (*p* = 0.001). However, no significant differences were observed among the groups regarding milk quality indicators (*p* > 0.05).

### 3.2. Plasma Biochemical Indices

Table 3 demonstrates that the inclusion of Zn-AA and Zn-Met have no significant difference relative to the CON group in plasma biochemical indices.

### 3.3. Antioxidant

Table 4 demonstrates that the levels of GSH-PX in the Zn-AA and Zn-Met groups were significantly higher than those in the control group (*p* = 0.003). Additionally, the inclusion of Zn-AA led to a significant rise in CAT content compared to the control group (*p* = 0.001), indicating that supplement with Zn-AA and Zn-Met can enhance the antioxidant ability for dairy cows. The levels of MDA in both Zn-AA and Zn-Met showed a decrease in comparison to the control group, but have no significant difference.

### 3.4. Immune Function

Table 5 demonstrates that the IgA content was significantly higher in the Zn-AA and Zn-Met groups compared to the control group (*p* = 0.001). Furthermore, the IgM content in the Zn-AA group was significantly elevated compared to the control group (*p* = 0.013).

### 3.5. Inflammatory Factor

Table 6 revealed no substantial variances in the levels of TNF-α, IL-1β, and IL-6 in plasma across all experimental groups.

### 3.6. Zn Content in Milk

**Figure 1:**
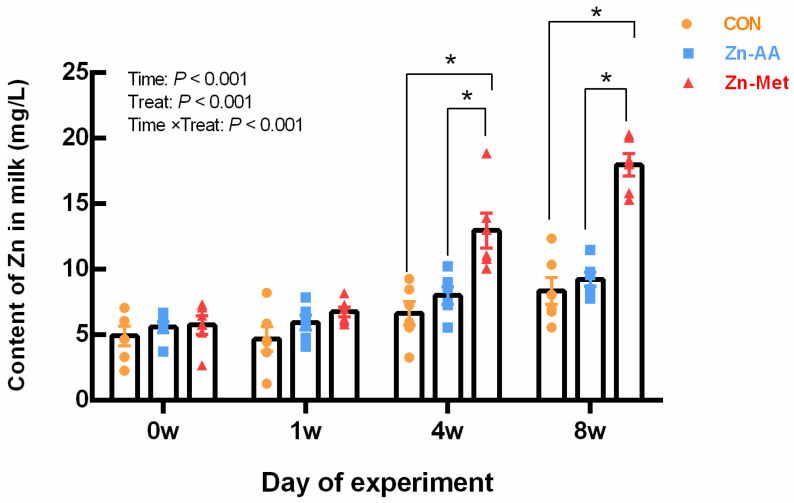
Effects of different diets on Zn content in milk of lactating cows. * The difference between the data representing the treatment group and that of the control group was significant (*p* < 0.001).

Compared with the control group, Zn-Met significantly increased (*p* = 0.001) the content of Zn in milk in the four weeks and eight weeks (Figure 1).

## *4.* Discussion

### 4.1. Effects of Different Zn Sources on Production Performance of Lactating Dairy Cows

The inclusion of 10 lactating cows in each treatment group in this study, while consistent with the sample size range of similar exploratory studies [31] may limit the statistical power to detect subtle effects, particularly those with high variability. Future confirmatory studies should expand the sample size to enhance the robustness of the conclusions.

The study concluded that the addition of ZnSO_4_, Zn-AA, and Zn-Met to the diet did not significantly impact the feed intake of dairy cows. Wang et al. [17] also reported that Zn supplementation in the basal diet did not affect the DMI of dairy cows. In contrast, Reza Alimohamady [32] found that Zn supplementation in the basal diet had a significant effect on DMI in lambs. The differences in results may be constrained by factors such as an insufficient sample size, species variation, production stage, zinc levels and sources, and the duration of supplementation [33]. Therefore, it is advisable to conduct further studies with a larger sample size study to verify these findings.

Zn supplementation from both inorganic and organic sources exhibits varying effects on milk production. The average milk yield of cows in the Zn-AA and Zn-Met groups showed a slight increase compared to the control group (CON); however, these differences were not statistically significant. The observed variations in milk production may be attributed to differences in the chelation strength of the organic Zn sources. Notably, a downward trend in milk fat content was observed in the Zn–Met group, potentially linked to the slightly higher milk production associated with Zn–Met compared to the control group. This finding aligns with the work of Salama et al. [34], who reported that the inclusion of Zn–Met in the diet resulted in an 8.8% reduction in milk fat concentration. This decrease in milk fat content may be related to the impact of Zn deficiency on the normal metabolic processes of fatty acids in dairy cows.

The results of this study indicate that the SCC in dairy cows is below the threshold of 200,000 cells/mL, as reported by Schwarz et al. [35]. However, Mishra et al. [36] noted that milk samples from healthy animals often contain few or nearly no somatic cells. Somatic cells can serve as indicators of an animal’s resistance and susceptibility to mastitis, facilitating the monitoring of subclinical mastitis levels in both groups and individual animals. A study by Overton and Yasui [37] and Salama et al. [34] demonstrated that Zn supplementation in dairy cows significantly reduced the number of somatic cells in milk, with SCC being notably lower in the organic Zn group compared to the control group. Furthermore, research by Yifan et al. [38] and Riad [39] supports the beneficial effects of Zn on udder health. In conclusion, the inclusion of organic zinc in the diets of lactating dairy cows has been shown to more effectively reduce SCC counts compared to inorganic zinc. This disparity may be attributed to the superior bioavailability of organic zinc, which optimizes zinc cell homeostasis by regulating the expression of zinc transporters, such as ZIP4. This regulation plays a crucial role in maintaining the integrity of the mammary epithelial barrier and enhancing the functional status of the metabolic regulatory network.

### 4.2. Effects of Different Zn Sources on the Health Status of Lactating Dairy Cows

Concentrations of blood biochemical parameters serve as indicators of nutritional status and the adequacy of the body’s nutrient supply [40]. ALT, AST, and γ-GT are significant transaminases in ruminants, closely associated with protein metabolism and liver-related functions [36]. Mandal et al. [41] reported no significant increase in AST, ALT, and γ-GT levels when comparing the effects of organic Zn to inorganic Zn in calves. Similarly, in this experiment, organic Zn supplementation had a minimal impact on these transaminases, indicating that different sources of Zn did not substantially affect the liver function of dairy cows. INS plays a crucial role in regulating the metabolism of proteins, sugars, and fats, while CHE is essential for the health and physiological functions of dairy cows [42]. Urea is critical for protein metabolism and serves as an indicator of renal function [43]. The observed increase in CHE, INS, and urea levels may be attributed to the high bioavailability and absorption rate of organic Zn.

### 4.3. Effects of Different Zn Sources on Antioxidant Indicators in Lactating Dairy Cows

Supplementation with organic Zn significantly enhances the activities of GSH-PX and CAT [6,44], aligning with the findings of this study. Furthermore, Zhu et al. [45] demonstrated that organic Zn supplementation reduces MDA levels and increases SOD activity in the serum of broiler chickens, which corroborates our results indicating that organic Zn is more beneficial than inorganic Zn in dairy cow feed. Compared to inorganic Zn, the supplementation of Zn–AA and Zn–Met resulted in an increase in SOD content, while simultaneously decreasing MDA content. Additionally, studies by Manimaran et al. [46] and Nagalakshmi et al. [47] reported increased GSH-PX and CAT activities in dairy cows and buffaloes fed organic Zn compared to those receiving inorganic sources. Overall, the effects of organic Zn on the antioxidant capacity of lactating dairy cows primarily involve the elevation of antioxidant enzyme levels and the scavenging of free radicals, collectively strengthening the antioxidant defense mechanism. These findings underscore the superior antioxidant capacity of organic Zn when compared to its inorganic counterpart.

### 4.4. Effects of Different Zn Sources on Immune Function of Lactating Dairy Cows

Immunoglobulins are essential for protective functions and serve as important indicators of human immune function [48]. Zn functions as an immune stimulant, enhancing both cellular and humoral immune responses [49]. By regulating the differentiation, proliferation, and activation of signaling pathways in immune cells, zinc enhances both cellular and humoral immune responses. At the molecular level, zinc promotes the differentiation of B lymphocytes into plasma cells by stabilizing the activity of transcription factors such as NF-κB and STAT, thereby improving the efficiency of antibody synthesis, including the production of IgG, IgM, and IgA [50,51]. The IgG, the primary antibody produced by the humoral immune response, plays a vital role in combating infections by neutralizing toxins and regulating the body’s defense mechanisms [52]. The IgM is the first antibody to appear in the body’s immune response to infections. The rapid generation of zinc is contingent upon the activation of the B-cell receptor (BCR) signaling pathway, as well as the facilitation of DNA replication by zinc-dependent enzymes, such as thymidylate synthase [53](Snijders et al. 2019). The IgA, the main antibody found in exocrine fluids, is secreted and generated by B lymphocytes [54]. Significantly higher concentrations of IgA and IgM were observed in the groups supplemented with Zn-AA and Zn-Met compared to the control group. Molecular-level analysis suggests that organic zinc may enhance the functionality of antigen-presenting cells (APCs), particularly by increasing the expression of MHC class II molecules on the surface of dendritic cells. This enhancement promotes collaboration between T cells and B cells, which activates B-cell class switching and increases the secretion of IgA and IgM.These results align with previous studies by Zhao [14] and Nagalakshmi et al. [47], which demonstrated that the addition of organic Zn to calf diets led to a notable increase in IgA and IgM levels, thereby strengthening immunity. This suggests that organic Zn can enhance the immune function of dairy cows and underscores its superior bioavailability compared to inorganic Zn [32].

### 4.5. Effects of Different Zn Sources on Inflammatory Factors in Lactating Dairy Cows

Zn homeostasis and Zn signaling are critical in regulating anti-inflammatory factors [55]. Monocytes and macrophages produce pro-inflammatory cytokines, including IL-1β, IL-6, and TNF-α [56]. Notably, TNF-α exhibits cytotoxic effects on tumor cells and also stimulates the secretion of IL-1β and IL-6 [57]. IL-6 enhances the inflammatory response, whereas IL-1β is recognized as a key regulator of inflammation [58,59]. This study observed no significant differences in the levels of IL-1β, TNF-α, and IL-6 across the various treatment groups, indicating that the source of supplemental Zn did not influence these cytokine levels.

### 4.6. Effects of Different Zn Sources on Zn Content in Lactating Dairy Cows

Numerous studies have examined the impact of Zn from various sources on Zn levels in milk, revealing varying concentrations [60,61]. The concentration of Zn in milk can be influenced by several factors, including the amount and form of Zn added to the diet, as well as individual cow characteristics. Research conducted by Novi [28] found that supplementing Zn to lactating dairy cows did not affect milk Zn concentration. Conversely, a study by Jie Cai et al. [62] demonstrated that cows fed Zn–methionine and nano-Zn had higher Zn concentrations in milk. These findings suggest that organic Zn may be more efficiently absorbed and utilized by dairy cows, leading to improved physiological support and increased Zn content in milk. Overall, organic Zn appears to positively impact the health and production performance of dairy cows.

## 5. Conclusions

This study compares the effects of zinc sulfate (CON), Zn-Met and Zn-AA on the physiological functions of dairy cows during early lactation. The results indicate that organic zinc sources do not significantly enhance DMI or milk yield compared to inorganic zinc sources. However, they exhibit specific biological effects: Zn-AA and Zn-Met significantly improved antioxidant and humoral immune functions by enhancing serum antioxidant capacity and increasing immunoglobulin (IgA and IgM) levels. Furthermore, Zn-AA significantly reduced the SCC in milk, indicating its regulatory effect on mammary gland inflammation. Remarkably, Zn-Met significantly increased the content of Zn in milk, suggesting that orzinc sources from Zn-Met can be absorbed by dairy cows, and further protect the mammary gland and reduce the mastitis. In conclusion, dietary supplementation with Zn-AA and Zn-Met positively influences the health of dairy cows in early lactation.

## Figures and Tables

**Table 1:** Nutritional composition and chemical composition of lactating dairy cows throughout the experiment.

Items	Treatments
Ingredient, % DM basis	
Alfalfa	8.39
Oat	6.43
Whole corn silage	52.18
Corn	8.1
Soya bean meal	10.8
Cotton seed meal	6.8
DDGS ^1^	4.8
Premix ^2^	2.5
NEL balance (Mcal/kg)	1.7
Crude protein (%)	17.07
Neutral detergent fiber (%)	34.94
Acid detergent fiber (%)	25.63
Ether extract (%)	3.85
Ash (%)	7.03
Ca (%)	0.88
P (%)	0.41
Zn (mg/kg DM)	60.00

^1^ DDGS: Dried corn alcohol grains. ^2^ Premix contains (per kg of DM): 1,100,000 IU of vitamin A, 270,000 IU of Vitamin D3, 200,000 mg of vitamin E, 4500 mg of vitamin K3, 300 mg beta-carotene, 60 mg of Cu, 100 mg of Fe, 85 mg of Mn, 20 mg of Co, 10 mg of Se, and 39 mg of I.

**Table 2:** Effects of Zn-AA and Zn-Met on production performance in early lactating cows.

Item	Treatment			SEM	*p*-Value
	CON	Zn-AA	Zn-Met		
DMI (kg/d)	24.92	25.07	25.03	0.30	0.523
Milk yield (kg/d)	38.67	38.89	38.78	0.29	0.105
FCR ^1^	1.54	1.43	1.56	0.06	0.176
4% FCM ^2^	39.28	37.94	39.38	2.24	0.282
Milk protein (%)	3.23	3.37	3.35	0.24	0.414
Milk fat (%)	4.21	4.24	4.23	0.29	0.973
Milk lactose (%)	5.12	5. 09	5.12	0.03	0.945
Total solids (%)	13.86	13.40	14.04	0.23	0.517
SCC (103/mL)	164.48 ^a^	118.33 ^c^	135.52 ^b^	28.00	0.001
MUN (mg/dL)	12.85	12.51	13.40	0.20	0.199

^1^ FCR = Feed conversion ratio. ^2^ 4%FCM = 4% Fat-corrected milk. The same letter or no letter in the shoulder label of the peer data had no significant difference (0.05 < *p* < 0.1), and different letters had significant difference (*p* < 0.05).

**Table 3:** Effects of Zn-AA and Zn-Met on plasma biochemical in early lactating cows.

Item	Treatment			SEM	*p*-Value
	CON	Zn-AA	Zn-Met		
Glu(mmol/L)	3.19	3.41	2.97	0.69	0.372
TG (mmol/L)	0.10	0.09	0.07	0.03	0.077
NEFA (mmol/L)	0.20	0.21	0.31	0.16	0.310
AST (U/L)	72.92	86.94	74.17	6.47	0.624
ALT (U/L)	25.58	29.17	29.08	1.34	0.496
ALP (U/L)	50.47	50.71	52.94	3.43	0.953
γ-GT (U/L)	28.09	31.84	26.47	1.94	0.509
TP (g/L)	62.67	63.09	60.77	2.24	0.910
ALB (g/L)	29.02	30.55	28.21	1.11	0.699
GLO (g/L)	33.65	32.54	32.56	1.43	0.941
TBIL (mmol/L)	2.62	2.74	2.34	0.53	0.057
CHE (U/L)	74.71	87.18	75.87	6.83	0.704
CREA-S (µmol/L)	56.15	62.61	58.53	2.42	0.561
UA (µmol/L)	37.36	41.44	36.39	1.92	0.514
UREA (mmol/L)	3.10	3.99	3.48	0.99	0.133
TC (mmol/L)	4.90	5.34	5.08	0.21	0.699
INS (μIU/mL)	3.55	5.02	3.94	0.47	0.434
Plasma Zn (µmol/L)	11.83	13.71	12.72	0.41	0.408

**Table 4:** Effects of different diets on plasma antioxidant lactating cows.

Item	Treatment			SEM	*p*-Value
	CON	Zn-AA	Zn-Met		
TAOC (mM/L)	3.82	3.90	3.72	0.38	0.574
GSH-PX (U/mL)	584.67 ^c^	789.39 ^a^	759.99 ^b^	134.10	0.003
SOD (U/mL)	96.09	120.39	113.91	30.96	0.185
CAT (U/mL)	110.72 ^b^	151.55 ^a^	70.69 ^c^	46.67	0.001
MDA (μmol/mL)	85.98	73.54	69.46	20.54	0.173

The same letter or no letter in the shoulder label of the peer data had no significant difference (0.05 < *p* < 0.1), and different letters had significant difference (*p* < 0.05).

**Table 5:** Effects of different diets on plasma immune lactating cows.

Item	Treatment			SEM	*p*-Value
	CON	Zn-AA	Zn-Met		
IgA (g/L)	8.10 ^c^	10.60 ^b^	12.78 ^a^	4.94	0.001
IgG (g/L)	48.60	52.90	53.10	5.04	0.924
IgM (g/L)	5.00 ^c^	6.32 ^a^	5.65 ^b^	1.05	0.013

The same letter or no letter in the shoulder label of the peer data had no significant difference (0.05 < *p* < 0.1), and different letters had significant difference (*p* < 0.05).

**Table 6:** Effects of dietary supplementation with different Zn sources on plasma inflammatory factors.

Item	Treatment			SEM	*p*-Value
	CON	Zn-AA	Zn-Met		
TNF-α (ng/mL)	0.61	0.55	0.59	0.04	0.563
IL-1β (ng/mL)	1.01	1.15	1.10	0.07	0.736
IL-6 (ng/mL)	0.89	0.82	0.93	0.07	0.798

## Data Availability

All data generated or analyzed during this study are included in this published paper.
